# Tirzepatide increased force of contraction in the isolated human atrium

**DOI:** 10.1007/s00210-025-04214-8

**Published:** 2025-04-29

**Authors:** Joachim Neumann, Britt Hofmann, Uwe Kirchhefer, Ulrich Gergs

**Affiliations:** 1 Institute for Pharmacology and Toxicology, Medical Faculty, Martin Luther University Halle-Wittenberg, Magdeburger Straße 4, 06097 Halle (Saale), Germany; 2 Department of Cardiac Surgery, Mid-German Heart Centre, University Hospital Halle, Ernst-Grube Straße 40, 06097 Halle (Saale), Germany; 3 Institute for Pharmacology and Toxicology, Medical Faculty, University Münster, Domagkstraße 12, 48149 Münster, Germany

**Keywords:** Glucose-dependent insulinotropic polypeptide receptor, Glucagon-like peptide-1 receptor, Tirzepatide, Human atrium

## Abstract

Tirzepatide is an approved drug that is used to treat type 2 diabetes. Tirzepatide is a peptide comprised of 39 amino acids and activates glucose-dependent insulinotropic polypeptide receptors (GIPR) and glucagon-like peptide-1 receptors (GLP-1R). Via GIPR and GLP-1R, tirzepatide stimulated in cell culture adenylyl cyclases (AC) and thereby elevated the cellular content of 3′:5′ cyclic adenosine monophosphate (cAMP). We tested the hypothesis that tirzepatide augmented the force of contraction (FOC) in isolated electrically driven (1 Hz) human right atrial preparations (HAP) obtained during open heart surgery from adult patients. Cumulatively applied tirzepatide, starting at nanomolar concentrations, raised FOC in a concentration-dependent manner and a time-dependent manner (*p* < 0.05). The positive inotropic effects (PIE) of tirzepatide were attenuated by about a quarter by a GIPR antagonist (100 nM, Pro3-GIP) and by about three quarters by a GLP-1R antagonist (100 nM, exendin9-39) in HAP. Tirzepatide (1 µM) was less effective than 1 µM isoprenaline in raising FOC in HAP. The inhibitor of the cAMP-dependent protein kinase called H89 reversed the PIE of tirzepatide. We suggest that tirzepatide probably acts via stimulation of GIPR and GLP-1R to exert a PIE in HAP.

## Introduction

**Fig. 1:**
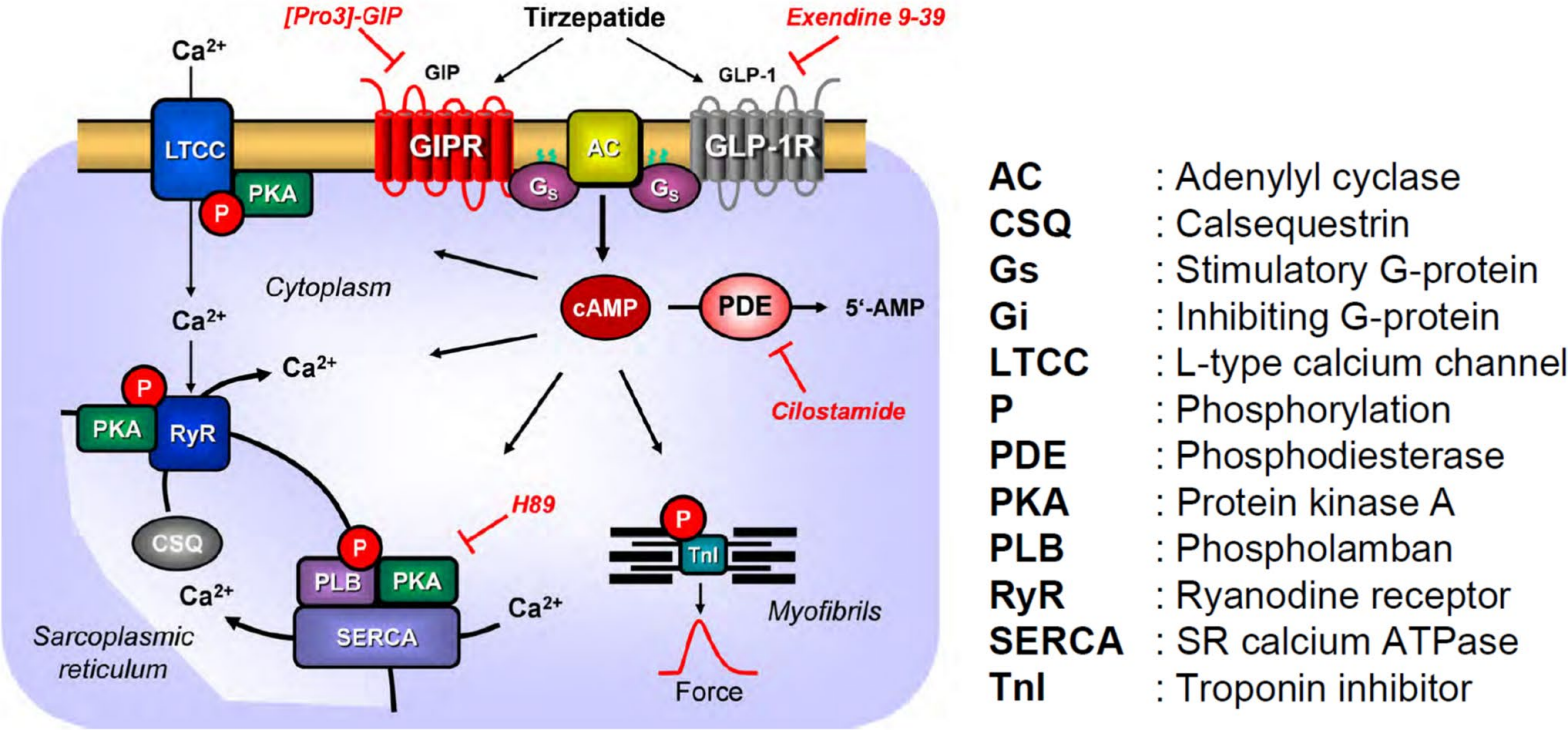
Putative mechanism(s) of action of tirzepatide in cardiomyocytes. Tirzepatide, like glucagon-like peptide-1 (GLP-1) itself stimulates GLP-1 receptors (GLP-1R). In addition, tirzepatide can be like glucose-dependent insulinotropic polypeptide (GIP) and also stimulate GIP receptors (GIPR). Then, via stimulatory GTP-binding proteins (Gs), adenylyl cyclases (AC) catalyze the formation of 3′:5′ cyclic adenosine monophosphate (cAMP). This cAMP activates a cAMP-dependent protein kinases (PKA). The cAMP is degraded by phosphodiesterase III (PDE), which can be inhibited by cilostamide. PKA can phosphorylate (encircled P), e.g., the inhibitory subunit of troponin (TnI) and phospholamban (PLB) which reduces the Ca^2+^ sensitivity of the myofilaments or enhances the action of SERCA, respectively. SERCA pumps Ca^2+^ into the sarcoplasmic reticulum. Ca^2+^ binds to calsequestrin (CSQ). RyR indicates the ryanodine receptor and is phosphorylated at least in part by PKA. LTCC means the L-type Ca^2+^ channel. Myofibrils are responsible for the generation of force, which is symbolized here by a single muscle contraction over time

Diabetes mellitus type 2 is critically connected to elevated blood glucose levels often accompanied by obesity (Drucker 2022, Forzano et al. 2022). Diabetes type 2 and obesity are risk factors for cardiovascular disease, notably for hypertension and for heart failure (Coskun et al. 2018). The release of insulin and glucagon in the pancreas can be regulated by various receptors (Holst 2007). As concerns expression in human organs, glucose-dependent insulinotropic polypeptide receptors (GIPR) and glucagon-like peptide-1 receptors (GLP-1R) are not only present in the human pancreas and mouse pancreas but also in the human heart and the mouse heart (Ussher et al. 2018). As concerns signal transduction in the heart, GIPR and GLP-1R can increase cAMP levels not only in the pancreas but also in the heart (Fig. 1, Heimbürger et al. 2020, Drucker 2022).

Tirzepatide is an approved drug that is used to treat type 2 diabetes (Jastreboff et al. 2022, Nauck and D’Alessio 2022). Currently, there are 93 ongoing or finished clinical studies in clinicaltrials.gov. Indications in these studies range from weight reduction, through arteriosclerosis, kidney disease, and alcoholism to the treatment of heart failure. A successful clinical trial in chronic diastolic heart failure has been recently published (Packer et al. 2024), underscoring the potential interest for tirzepatide in cardiology. Tirzepatide exhibits a modified protein sequence compared to the endogenous human peptides GLP-1 and GIP (Forzano et al. 2022). Tirzepatide reduced blood glucose levels and led to weight loss in humans, and therefore, tirzepatide was used successfully against obesity in humans (Jastreboff et al. 2022, review: Forzano et al. 2022).

Tirzepatide has a sequence of 39 amino acids. Tirzepatide contains non-conventional amino acid residues at positions two and 13 (α-amino isobutyric acids), and the C-terminus is amidated (Coskun et al. 2018). Tirzepatide at amino acid 20, lysine, contains a fatty acid moiety that prolongs the half-life of tirzepatide (Syed 2022). Tirzepatide acts as an agonist with high affinity (similar to endogenous GIP) at GIPR and binds with lower affinity at GLP-1R than endogenous GLP-1 (Coskun et al. 2018). Tirzepatide has about a 40-fold higher affinity for GIPR than for GLP-1R (Coskun et al. 2018). Hence, one might hypothesize that, if we add tirzepatide to human right atrial preparations (HAP), we stimulate mainly GIPR and to a lesser extent GLP-1R in HAP. Conceivably, tirzepatide might also stimulate only GIPR or only GLP-1R or neither in HAP.

We initiated the present study to find out whether tirzepatide, like the cAMP-increasing agent isoprenaline (Fig. 1), can affect the mechanical function of the human heart. A progress report of the present work has been published in abstract form (Neumann et al. 2024 d).

We tested mainly the following hypotheses:

1. Tirzepatide increases the force of contraction (FOC) in HAP.
2. This positive inotropic effect (PIE) is only mediated by GIPR.
3. This PIE is only mediated by GLP1-R.

## Materials and methods

### Contractile studies on human preparations

The contractile studies on HAP were performed using the same setup, and a modified Tyrode’s solution was not altered compared to our previous studies (e.g., Gergs et al. 2009, 2017). In brief, human right atrial preparations obtained during the cardiac surgery at the sites where extracorporeal circulation needles were inserted were transferred within 30 min into the laboratory in the modified Tyrode’s solution. Samples were cut into small trabecular muscle pieces. These muscle strips were then mounted under isometric conditions with metal hooks at each end of the muscle in a glass organ bath. Human muscle strips were stretched to the maximum of the force-contraction relationship. The HAP obtained from 15 patients (four females and 11 males), aged between 62 and 83 years, was acquired. From each patient’s right cardiac appendage, we obtained muscle strips as delineated in the figure legends. The patients suffered from severe coronary diseases (two and three vessel diseases). The cardiac drug therapy included acetylsalicylic acid, apixaban or a similar anticoagulant, furosemide or a similar a loop diuretic, and bisoprolol or a similar β-adrenoceptor antagonist. Cardiac comorbidities included angina pectoris, hypertension, and atrial fibrillation.

The modified Tyrode’s solution contained in millimolar concentrations (mM): 119.8 NaCI, 5.4 KCl, 1.8 CaCl_2_, 1.05 MgCl_2_, 0.42 NaH_2_PO_4_, 22.6 NaHCO_3_, 0.05 Na_2_EDTA, 0.28 ascorbic acid, and 5.05 glucose. Ascorbic acid is used here as an antioxidant to maintain the activity of, for instance, isoprenaline. The solution was continuously gassed with 95% O_2_ and 5% CO_2_ and maintained at 37 °C and pH 7.4 in the organ baths. HAP were stimulated (60 beats per minute, bpm) electrically with platinum electrodes with rectangular impulses of direct currents from a Grass stimulator SD 9 (Quincy, USA). Voltage ranged between 5 and 10 V, just sufficient to initiate contractions. The drugs tested failed to impact the pacing threshold. Electrical impulses had a length of 5 ms. The signals from the force transducer were fed into a bridge amplifier, digitized, and stored on a commercial personal computer. The signals were quantified using a commercial software (Lab Chart 8 from ADInstruments bought through their distributor in Oxford, England).

### Data analysis

Data shown are mean ± standard error of the mean. Statistical significance was estimated using a Student’s *t*-test or the analysis of variance followed by Bonferroni’s *t*-test as described in the legends. A *p*-value < 0.05 was considered to be significant.

### Drugs and materials

(−)Isoprenaline tartrate (dissolved in water) and H89 (dissolved in dimethylsulfoxide) were from Sigma-Aldrich (Taufkirchen, Germany). Tirzepatide was from Biozol (Eching, Germany, dissolved in water). Pro3GIP (ProGIP) and exendin9-39 (exendin) came from Bachem (Bubendorf, Switzerland, both dissolved in water). All other chemicals were of the highest purity grade commercially available. Deionized water was used throughout the experiments to prepare a modified Tyrode’s solution. Stock solutions were prepared fresh daily.

## Results

**Fig. 2:**
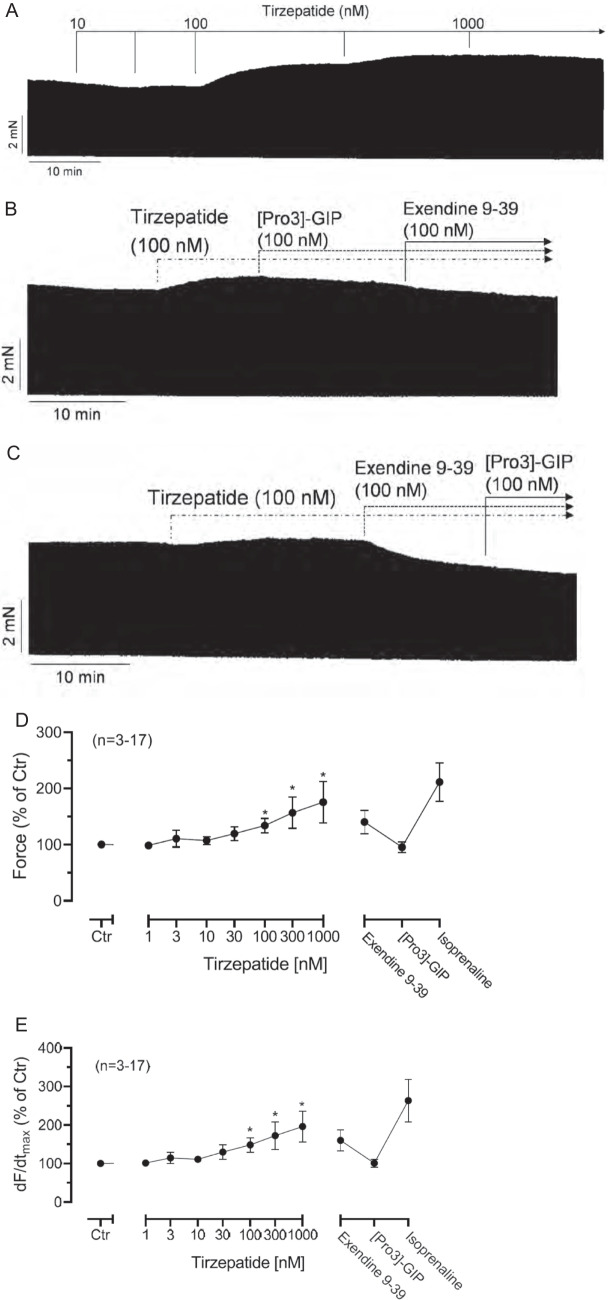

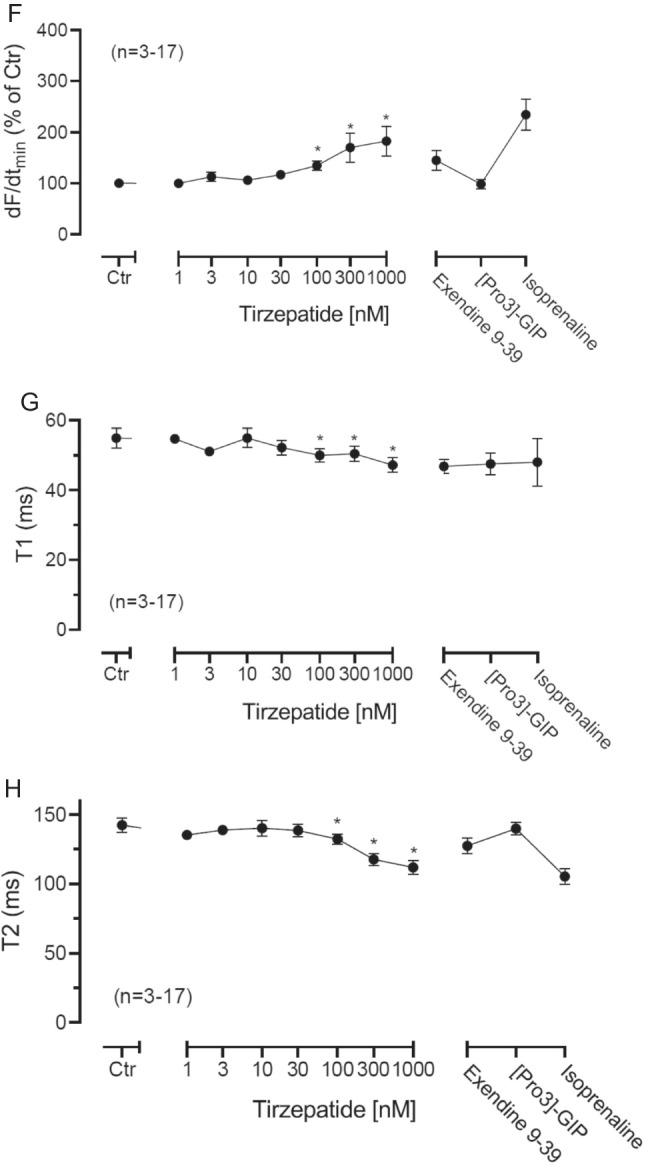
Tirzepatide alone induced a PIE in HAP. **A** Original recordings in human right atrial preparations. Concentration-dependently, tirzepatide alone induced a time-dependent positive inotropic effect in HAP. Above the figure, the addition of tirzepatide was indicated with an arrow and vertical lines. Left vertical bar: FOC in mN. Lower horizontal bar: time in minutes (min). Upper horizontal bar indicates cumulative addition of tirzepatide at the indicated concentration in nanomoles per liter (nM). **B** Original recording in human right atrial preparations. Tirzepatide (100 nM) induced a time-dependent positive inotropic effect in HAP. The addition of tirzepatide was indicated with broken arrows. Additions of Pro3-GIP and then additionally exendin (9–39) are indicated with horizontal arrows (top). Left vertical bar: FOC in mN. Lower horizontal bar: time in minutes (min). Upper horizontal bar indicates cumulative addition of tirzepatide at the indicated concentrations in nanomoles per liter (nM). **C** Original recording in human right atrial preparations. Tirzepatide (100 nM) induced a time-dependent positive inotropic effect in HAP. The addition of tirzepatide was indicated with broken arrows. Additions of exendin (9–39) and subsequently Pro3-GIP are indicated with horizontal arrows (top). Left vertical bar: FOC in mN. Lower horizontal bar: time in minutes (min). Upper horizontal bar indicates cumulative addition of tirzepatide at the indicated concentration in nanomoles per liter (nM). Summarized effects of tirzepatide on force of contraction (**D**), rate of tension development (**E**, dF/dt_max_), and rate of tension relaxation (**F**, dF/dt_min_), on time to peak tension (T1, **G**), and time to relaxation (**H**). Ctr (pre-drug value), *p < 0.05 vs. Ctr. The number in brackets indicates the number of muscle strips (HAP) in these experiments and came from three to seven patients

In HAP, a cumulatively applied tirzepatide *alone* increased FOC within minutes of incubation starting at 100 nM and reached a plateau at 1 µM. This is shown in a typical experiment (Fig. 2A). As is typical for cAMP-increasing agents like isoprenaline, tirzepatide increased the rate of tension development and the rate of tension relaxation in HAP (Fig. 2B, C). Like isoprenaline, tirzepatide shortened the time to peak tension and the time of relaxation in HAP (Fig. 2D, E). Additionally applied, 100 nM exendin9-39 reduced the FOC of HAP that had been raised by tirzepatide (Fig. 2B), suggesting that the PIE of tirzepatide is in large part due to GLP-1R. Moreover, Pro3GIP, an antagonist at GIPR, when given first after tirzepatide application, decreased the force of contraction, suggesting that the PIE of tirzepatide was mediated in minor part by GIPR (Fig. 2B). When one quantified these relative contributions, we calculated that the effect of tirzepatide was inhibited through ProGIP by 23.7 ± 6.6% and inhibited by exendin by 76.3 ± 6.6% (*n* = 5, each). Moreover, 10 µM propranolol, a concentration we used before to block the contractile effect of the release of noradrenaline by, e.g. amphetamine (Neumann et al. 2023), did not abolish the PIE of tirzepatide. In the presence of 10 µM propranolol, 100 nM tirzepatide increased the force of contraction in HAP to 204 ± 9.7% compared to a pre-drug (tirzepatide) value (*n* = 3, *p* < 0.05).

**Fig. 3:**
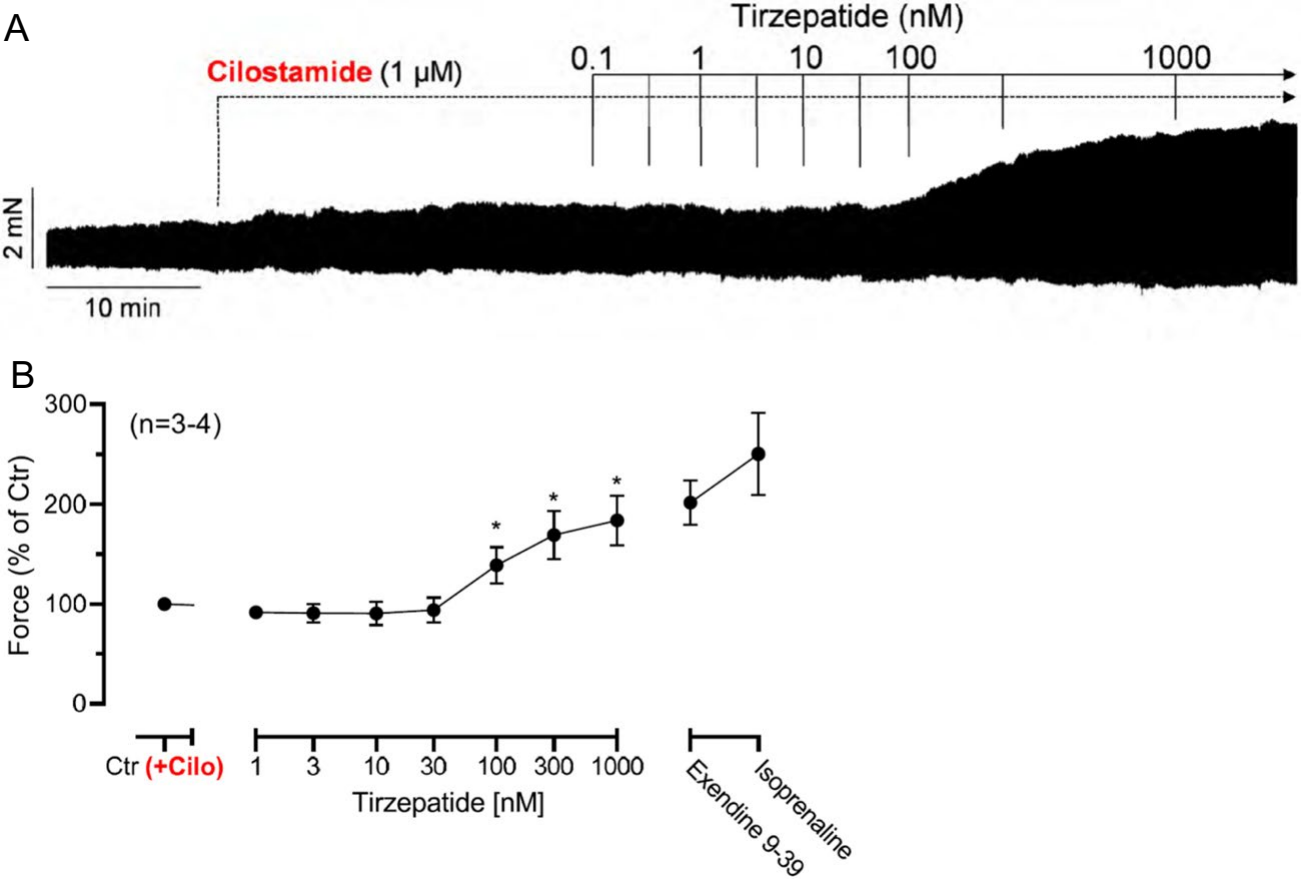
Tirzepatide in the presence of cilostamide exerts a PIE in HAP. **A** Original recording in human right atrial preparations. First, cilostamide was given to increase FOC slightly. Then, increasing concentrations of tirzepatide induced a time-dependent positive inotropic effect in HAP. Left vertical bar: FOC in mN. Lower horizontal bar: time in minutes (min). Upper horizontal bar indicates cumulative addition of tirzepatide at the indicated concentration in nanomoles per liter (nM). Summarized effects of tirzepatide on the force of contraction (**B**), ordinates indicate force in milli-Newton. *p < 0.05 vs. Ctr (pre-drug value). The number in brackets indicates the number of muscle strips (HAP) in these experiments and came from four patients

When we gave first cilostamide, a PDE III inhibitor which we used with success in the HAP in separate contexts (Gergs et al. 2023, Neumann et al. 2004b) to the organ bath to slightly elevate the force of contraction and then added tirzepatide, we could raise FOC. This is depicted in a typical original experiment in Fig. 3A. Tirzepatide exerted, in the presence of 1 µM cilostamide, a concentration and time-dependent positive inotropic effect (Fig. 3B). Cilostamide had a tendency to increase the potency of tirzepatide, namely changing the negative decadic logarithm of EC50 values from 6.98 (*n* = 10) to 7.45 (*n* = 4), but this effect did not reach significance (*p* = 0.18). Moreover, the effect of tirzepatide could be attenuated by exendin9-39. Exendin is a truncated derivative of exenatide. Whereas exenatide is an agonist at GLP-1R and has a PIE in HAP, exendin 9–39 is an antagonist at exenatide and attenuates the PIE of exenatide at HAP (Wallner et al. 2015, Neumann et al. 2024b). Hence, the PIE of tirzepatide is antagonized by exendin and is therefore in part mediated by GLP-1R.

Next, the question arose whether the PIE of tirzepatide in HAP might also be due to a stimulation of cardiac GIPR. This was a reasonable hypothesis: tirzepatide binds with higher affinity at GIPR than at GLP-1P. Moreover, we ourselves had recently reported that GIP, as a peptide, can increase FOC in HAP (Neumann et al. 2024b, 2024c). The PIE of 100 nM GIP was enhanced by cilostamide in HAP and was completely reversed by 100 nM ProGIP (Neumann et al. 2024b, 2024c). ProGiP is a mutated form of GIP where amino acid three is mutated to proline. In this way, the agonist GIP is mutated to the antagonist ProGIP (Fig. 3A). Indeed, when we applied tirzepatide and then after the PIE had reached a plateau and when we then added ProGIP, we noted a NIE of ProGIP, suggesting that the PIE of tirzepatide was in part due to stimulation of GIPR (Fig. 3B).

Please note that when we summarized the effects of tirzepatide in the presence of cilostamide on the force of contraction, we usually noted that isoprenaline was more effective than tirzepatide (Fig. 3B). Compounds that increase the FOC via cAMP usually phosphorylate the ryanodine receptor (Fig. 1). This usually leads to an increase in the maximum rate of tension development. This was also noted with tirzepatide and supports our interpretation that tirzepatide acts at least in part via cAMP-dependent phosphorylation (Fig. 3C, dF/dt_max_). Moreover, cAMP-dependent phosphorylation in the heart usually increases the phosphorylation state of phospholamban (Fig. 1). This leads to an enhanced rate of uptake of calcium cations from the cytosol into the sarcoplasmic reticulum. Less calcium cations are thus located near the myofilaments and can generate force, therefore the maximum rate of tension relaxation (Fig. 5D, dF/dt_min_) time to peak tension (Fig. 3E) and time to relaxation rise. This was what we observed with tirzepatide, and this also supports the view that tirzepatide acts via cAMP in the human heart (Fig. 3F).

**Fig. 4:**
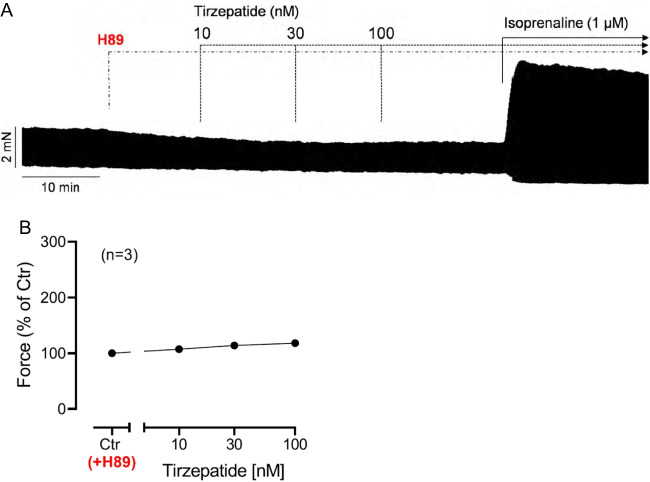
H89 reversed the PIE of tirzepatide in HAP. **A** Original recording in human right atrial preparations. Concentration-dependent effect of tirzepatide in the presence of 5 µM H89 in HAP. Ordinate indicates force of contraction in mN. **B** Summarized effects of tirzepatide on force of contraction. Ordinate gives force in percentage of pre-drug value = before 5 µM H89, *p < 0.05 vs. Ctr (pre-drug value). Number in brackets indicates the number of muscle strips (HAP) in these experiments and came from three patients

If the PIE of tirzepatide is mediated by a cAMP-dependent activation of the cAMP-dependent protein kinase (Fig. 1), then an inhibitor of the cAMP-dependent protein kinase, like H89, should attenuate the PIE of subsequently applied tirzepatide. This assumption held true. This can be noted in an original recording (Fig. 4A): treatment with H89 attenuated the subsequent PIE of tirzepatide. Several such experiments led to the same result, as was illustrated in Fig. 4B.

## Discussion

The main new finding is that tirzepatide alone exerts a PIE in HAP. This is clinically relevant because in May 2022, tirzepatide was approved in the USA to treat type 2 diabetes (Syed 2022). Tirzepatide is currently being tested in phase III studies for the treatment of heart failure (Syed 2022). Tirzepatide was beneficial in patients with diastolic heart failure (Packer et al. 2024).

The targets of tirzepatide, namely GIP and GLP-1, are often summarized as incretins. Oral food intake leads to the release of GIP and GLP-1 from their stores in the human intestinal tract (Syed 2022). GIP and GLP-1 enter the blood and stimulate GIPR and GLP-1R, respectively, in the pancreas (Syed 2022). Thus, they reduce the secretion of glucagon and increase the secretion of insulin from the pancreas (Syed 2022). Eight miligrams of tirzepatide led to peak plasma concentrations of around 180 nM (Coskun et al. 2018). This means that the PIE, we described in this report, occurs at concentrations that are similar to therapeutic plasma concentrations of tirzepatide. Also, on this basis, we argue that our findings occurring at drug concentrations are clinically relevant.

### Mechanism of action of tirzepatide

The PIEs of tirzepatide in HAP are probably dual-receptor-mediated. This conclusion is supported by the observation that the PIE of tirzepatide in HAP was attenuated by both a GIPR antagonist, Pro3GIP, and a GLP-1R antagonist, exendin9-39. Tirzepatide has been shown to increase cAMP in neonatal mouse cardiomyocytes (Hiromura et al. 2016). At least with exenatide, a GLP-1R-mediated protein phosphorylation in HAP has been reported (Wallner et al. 2015), and thus it is plausible to occur also with tirzepatide. To rule out that tirzepatide stimulated β-adrenoceptors or released noradrenaline, we performed separate experiments after pre-treatment with 10 µM propranolol. Under these conditions, the PIE of tirzepatide was still present, suggesting that tirzepatide is not an agonist at β-adrenoceptors and does not release noradrenaline from stores in the atrial preparations.

### Species differences

Of note, GIPR agonists and GIP-1R agonists alone failed to increase the beating rate in RA or FOC in LA (Neumann et al. 2024a, Baggio et al. 2017, respectively). However, in the presence of rolipram, GIP increased FOC but not the beating rate in LA or RA, respectively (Neumann et al. 2024a,b,d).

### Comparison to other studies

Others and ourselves reported that exenatide, a GLP-1R agonist, increased force of contraction in HAP (Wallner et al. 2015, Neumann et al. 2024a). We also reported that semaglutide and liraglutide, two more novel GLP-1R agonists, increased FOC in HAP (Neumann et al. 2024a). These reports agree that GLP-1R stimulation can raise force in HAP. Therefore, these data support our hypothesis that tirzepatide is an agonist at GLP-1R. We have recently reported that GIP increased FOC in HAP via GIPR (Neumann et al. 2024b,c).

### Clinical relevance

Using the highest dosage of tirzepatide (15 mg per week) in a recent study, a peak plasma concentration of about 2200 ng/ml (458 nM) of tirzepatide was reported (Furihata et al. 2022). Hence, the concentration of 100 nM tirzepatide that we used here is well within the therapeutic window of tirzepatide. Tirzepatide is an approved drug for the treatment of type 2 diabetes and is probably useful to treat obesity (vide supra). Of concerns regarding cardiac effects, they are likely to occur as the therapeutic drug concentration of tirzepatide in our conditions had a significant positive inotropic effect. It is uncertain whether these cardiac effects of tirzepatide in HAP are detrimental or beneficial for the patient. There are data that tirzepatide increased the heart rate of patients (Frías et al. 2021). This might be due to a cAMP elevation and subsequent stimulation of ion channels in the sinus node in patients, in contrast to our findings in RA. Alternatively, the PCE might be compensatory in humans: tirzepatide reduced blood pressure in humans, and this can lead via central mechanisms to a reflective tachycardia (Frías et al. 2021). A PIE might be symptomatically useful in heart failure patients. However, all cAMP-increasing drugs can lead to supraventricular and ventricular arrhythmias. These side effects should be kept in mind as a probable hazard. There are retrospective patient data that suggest that tirzepatide reduces the incidence of atrial fibrillation in obese patients (Baser et al. 2024). However, others regard this question as open and refer to ongoing prospective trials that have as a primary endpoint whether tirzepatide can reduce the incidence of arrhythmias in obese patients (Patoulias et al. 2022, e.g. NCT06802081).

### Limitations of the study

One can argue that we have not tested the effects on the sinus node of man directly, but only in mice. Hence, we may have overlooked a GLP-1R or GIPR-mediated PCE in the human heart. We did not have the opportunity to study contractility in the human ventricle tissue for lack of access to that tissue. Hence, it needs to be studied by others whether tirzepatide affects PIE in human ventricle strips. However, only in about 17% of human patients, GCP-R stimulation induced a PIE (Wallner et al. 2015). As far as we know, GIP-mediated inotropic effects in isolated human ventricular preparations have not yet been reported and should be studied first to clarify basic mechanisms in the human ventricle.

In summary, we can now address the hypotheses raised in the Introduction in this way: Tirzepatide exerts a PIE in HAP. This PIE is mediated to the smaller part by GIPR and to the larger part by GLP-1R.

## Data Availability

All source data for this work (or generated in this study) are available upon reasonable request.
